# Editorial: Metallic micronutrient homeostasis in plants, volume II

**DOI:** 10.3389/fpls.2023.1329190

**Published:** 2023-12-01

**Authors:** Hannetz Roschzttardtz, Diego Gomez-Casati, Christian Dubos, Julia Quintana

**Affiliations:** ^1^ Facultad de Ciencias Biológicas, Pontificia Universidad Católica de Chile, Santiago, Chile; ^2^ Centro de Estudios Fotosintéticos y Bioquímicos (CEFOBI-CONICET), Universidad Nacional de Rosario, Rosario, Argentina; ^3^ Institut des Sciences des Plantes de Montpellier (IPSiM), University Montpellier, CNRS, INRAE, Montpellier, France; ^4^ Center for Plant Biotechnology and Genomics, Universidad Politécnica de Madrid, Madrid, Spain; ^5^ Department of Biology and Geology, Physics and Inorganic Chemistry, Universidad Rey Juan Carlos, Madrid, Spain

**Keywords:** plant nutrition, metal accumulation, transcriptomics, seed nutrient composition, metal homeostasis, iron deficiency

“*Metallic Micronutrient Homeostasis in Plants, Volume II*” represents an initiative that responds to the need for a research area enriched with its unique historical context and challenges, all of which are critically relevant in the context of the ever-evolving global environmental landscape. The development and use of amenable techniques for metal localization *in vivo* remains a major experimental challenge in the study of metal nutrient homeostasis. Moreover, analysis of the interactions between micronutrient and macronutrient homeostasis is gaining more and more attention within the scientific community. Lastly, there is a pressing need to expand the knowledge in iron deficiency responses in non-model crops. This Research Topic agglutinates knowledge in these directions. In one of the contributions,

Grant-Grant et al. have elegantly demonstrated that alterations in the spatial distribution of iron within plant embryos do not exert a significant influence on the overall iron content within the seeds. This suggests that the regulation of iron levels within plants is not determined by how iron is distributed within the embryo. From a biotechnological perspective this discovery is interesting as it demonstrates the difficulty in designing strategies to increase micronutrients in seeds.

Xu et al., have used different maize inbred lines in order to identify potential important genes in iron deficiency tolerance in maize, which affect a significant proportion of cultivable land on the planet. This study provides valuable insights into unravelling the molecular mechanisms that underlie maize’s resilience to iron-deficiency stress.

In other work, Yuan et al. identified potential genes for maintaining iron homeostasis under iron-deficient stress in non-heading Chinese cabbage. This could provide a robust theorical foundation for the research on molecular mechanisms governing the adaptation to iron stress and could also serve to design new strategies for the development of iron-tolerant crop varieties.

How plants respond to iron deficiency stress is an important area of research in plant biology. Tran et al. demonstrated that the alteration of porphyrin biosynthesis via the introduction of the *Arabidopsis thaliana* Mg-chelatase H subunit gene (AtCHLH) into transgenic rice effectively mitigates stress induced by iron deficiency. It has been proposed the existence of an ABA-independent pathway as a potential mechanism for alleviating this stress through protoporphyrin synthesis.

In summary, the work presented in this Research Topic showed the importance of different genes in the distribution and the maintenance of iron homeostasis, specially under iron stress conditions. Interestingly, is has been proposed that regulatory mechanism for porphyrin metabolism would be part of the complex protective systems against Fe deficiency stress. Moreover, the landscape of iron deficiency transcriptomes has been enriched with an interesting analysis of gene expression changes in Chinese cabbage. All in all, this research aims to ultimately aid the development of new solutions to mitigate fertilizer overuse and while increasing crop nutritional value.

## Author contributions

HR: Writing – original draft. DG-C: Writing – original draft. CD: Writing – original draft. JQ: Writing – original draft.

